# Whole blood assessment of antigen specific cellular immune response by real time quantitative PCR: a versatile monitoring and discovery tool

**DOI:** 10.1186/1479-5876-6-58

**Published:** 2008-10-16

**Authors:** Elke Schultz-Thater, Daniel M Frey, Daniela Margelli, Nermin Raafat, Chantal Feder-Mengus, Giulio C Spagnoli, Paul Zajac

**Affiliations:** 1 Institute of Surgical Research and Hospital Management, Dept. of Biomedicine, University Hospital of Basel, Basel, Switzerland; 2 Personnel Medical Service, University Hospital of Basel, Basel, Switzerland

## Abstract

**Background:**

Monitoring of cellular immune responses is indispensable in a number of clinical research areas, including microbiology, virology, oncology and autoimmunity. Purification and culture of peripheral blood mononuclear cells and rapid access to specialized equipment are usually required. We developed a whole blood (WB) technique monitoring antigen specific cellular immune response in vaccinated or naturally sensitized individuals.

**Methods:**

WB (300 μl) was incubated at 37°C with specific antigens, in the form of peptides or commercial vaccines for 5–16 hours. Following RNAlater addition to stabilize RNA, the mixture could be stored over one week at room temperature or at 4°C. Total RNA was then extracted, reverse transcribed and amplified in quantitative real-time PCR (qRT-PCR) assays with primers and probes specific for cytokine and/or chemokine genes.

**Results:**

Spiking experiments demonstrated that this technique could detect antigen specific cytokine gene expression from 50 cytotoxic T lymphocytes (CTL) diluted in 300 μl WB. Furthermore, the high sensitivity of this method could be confirmed ex-vivo by the successful detection of CD8+ T cell responses against HCMV, EBV and influenza virus derived HLA-A0201 restricted epitopes, which was significantly correlated with specific multimer staining. Importantly, a highly significant (p = 0.000009) correlation between hepatitis B surface antigen (HBsAg) stimulated IL-2 gene expression, as detectable in WB, and specific antibody titers was observed in donors vaccinated against hepatitis B virus (HBV) between six months and twenty years before the tests. To identify additional markers of potential clinical relevance, expression of chemokine genes was also evaluated. Indeed, HBsAg stimulated expression of MIP-1β (CCL4) gene was highly significantly (p = 0.0006) correlated with specific antibody titers. Moreover, a longitudinal study on response to influenza vaccine demonstrated a significant increase of antigen specific IFN-γ gene expression two weeks after immunization, declining thereafter, whereas increased IL-2 gene expression was still detectable four months after vaccination.

**Conclusion:**

This method, easily amenable to automation, might qualify as technology of choice for high throughput screening of immune responses to large panels of antigens from cohorts of donors. Although analysis of cytokine gene expression requires adequate laboratory infrastructure, initial antigen stimulation and storage of test probes can be performed with minimal equipment and time requirements. This might prove important in "field" studies with difficult access to laboratory facilities.

## Introduction

Routine monitoring of immune responses is usually limited to the detection of humoral responsiveness and the capability of inducing adequate antibody titers represents the gold standard for virtually all vaccines of current use for the prevention of infectious diseases. In contrast, monitoring of cellular immune responses following natural or vaccine induced immunization is far less standardized. A number of different techniques have been developed. They include limiting dilution analysis of specific T cell precursors, multimer staining of antigen specific T cells, intracellular staining with cytokine specific antibodies, ELISPOT or ELISA assays for antigen driven cytokine production, antigen specific cytotoxicity and lymphoproliferation assays or quantitative real-time polymerase chain reaction (qRT-PCR) for the detection of cytokine gene expression [1-3].

These methods generally require gradient purification of peripheral blood mononuclear cells (PBMC), culture for different time periods in sterile CO2 incubators or rapid access to highly specialized lab equipment and the use of biologicals, e.g. FCS or human serum from different sources. Furthermore, professional skills are also required. As a result, monitoring of cellular immune responses is difficult to standardize, and a high variability of results from different laboratories is frequently observed, hindering the performance of multi centre comparative studies [4-6].

Detection of cytokine (CK) gene expression by quantitative RT-PCR (qRT-PCR) has been successfully applied to the monitoring of immune responses in PBMC [7], in tumor specimens [8,9] or to the identification of antigenic epitopes [10-12].

We sought to further develop these methods into a simple technique, easily amenable to automation, allowing accurate monitoring of antigen specific cellular immune responsiveness in whole blood (WB) of individuals undergoing vaccinations or naturally sensitized to specific antigens.

Similar techniques have been described in the past. However, most of these studies mainly focused on responsiveness to endotoxins, did not explore correlations with protection against infectious challenges or adequate surrogate markers, or addressed only a limited variety of genes thereby potentially failing to identify specific gene expression profiles associated with clinical manifestations [13-16].

Here we show that WB monitoring of cellular immune responses by qRT-PCR, represents a sensitive and specific method capable of efficiently unravelling gene expression profiles associated with vaccination or natural immunization.

## Materials and methods

### Reagents

Antigenic peptides encompassing HLA-A*0201 restricted human cytomegalovirus (HCMV) pp65_495–503_, Epstein-Barr virus (EBV) BMLF-1_259–267_, EBV LMP-2_426–434 _and influenza matrix (IM) _58–66 _virus derived epitopes [17,18] used to assess specific T cell responses were obtained from Neosystem (Strasbourg, France). Corresponding peptide specific PE labelled HLA-A*0201 multimers were from Proimmune (Abingdon, UK). Hepatitis B virus (HBV) (Engerix, Glaxo Smith Kline, Münchenbuchsee, Switzerland) and influenza (Inflexal, Berna Biotech, Bern, Switzerland) commercial vaccine preparations were used to monitor T-cell responses to vaccination.

### Cell cultures

PBMC were isolated from peripheral blood of healthy donors by Ficoll gradient centrifugation. When indicated, specific PBMC subpopulations were purified by magnetic cell separation (Miltenyi Biotech, Bergisch Gladbach, Germany) according to producers' protocols. Cells were then cultured in RPMI 1640 supplemented with 100 μg/ml Kanamycin, 10 mM Hepes, 1 mM sodium pyruvate, 1 mM Glutamax and non-essential amino acids (all from GIBCO Paisley, Scotland), thereafter referred to as complete medium, and 5% (v/v) human serum (Blutspendezentrum, University Hospital Basel, Switzerland).

For proliferation assays cells were cultured in presence of antigenic preparations or in the absence of stimuli in 96-well flat bottom tissue culture plates (Becton Dickinson, Le Pont de Claix, France), at 2 × 10^5 ^cells per well, in triplicates. On day six, cultures were pulsed with 1 μCi per well of [^3^H] thymidine (Amersham, Little Chalfont, UK) for 18 h and then harvested. Tracer incorporation was measured by β-counting.

### Phenotypic characterization of cells

PBMC were phenotyped by staining with FITC- or PE-conjugated mouse monoclonal antibodies (mAb) to human CD8 and CD4 (Becton Dickinson, San Diego, CA). CD8+ lymphocytes bearing specific T cell receptors were identified by staining with HLA-A0201 multimers containing the desired peptide (Proimmune, Oxford, UK) [19]. Data were reported as total number of MHC-multimer+/CD8+ cells obtained from volumes of WB equal to those utilized for RNA extraction.

### ELISA and Elispot assays

Antibody response to HBs Ag was evaluated by ELISA assays (Architect System, Abbott, Sligo, Ireland) in sera from naïve or vaccinated donors.

Elispot assays for the enumeration of IFN-γ or IL-2 producing cells were performed as described previously [20].

### WB monitoring of cellular immune responses

Appropriate concentrations of specific antigens, in the form of peptides or commercial vaccine preparations (see above) were added to 0.3 ml of heparinized peripheral blood in 2 ml tubes. Samples were then centrifuged for ten seconds in a minifuge to bring cells in close contact and incubated for 5 h or 16 h, for peptide or vaccine preparations, respectively, at 37°. Three volumes of RNAlater (Ambion, no. AM7020, Austin TX) were then added to stabilize RNA. The mixture was then either stored at different temperatures (see below) or treated immediately for RNA extraction. Sterile hoods, incubators or ≤-20°C refrigerators were not required.

### RNA processing and Real Time PCR

Total cellular RNA was extracted by using Ribo Pure-Blood kit (Ambion Inc., no. AM1928, Austin, TX, USA) and eluted in 75 μl of elution buffer. Reverse transcription was done with 11 μl of total RNA by priming it with 1 μl (200 μg/ml) of Oligo dT (Roche Diagnostics, Mannheim, Germany) at 65°C for 10 minutes and quick chilling on ice. This mixture was supplemented with 1 μl 10 mM dNTP mix, 4 μl 5× first-strand buffer, 2 μl 0.1 M DTT and 1 μl (200 units) M-MLV reverse transcriptase (all by Invitrogen Ltd., Paisley, UK) and incubated at 37°C for 1 hour. Two μl of cDNA were used for each PCR amplification by "real time" technology (7300 Real Time PCR system, Applied Biosystems, Rotkreuz, Switzerland) according to manufacturer's recommendation in the presence of primers and probes specific for genes encoding IFN-γ, IL-2, IL-6, IL-10 and TNF-α as already described [21] or MIP-1β (Assays-on-demand, Applied Biosystems, Rotkreuz, Switzerland). Antigen driven cytokine gene expression (triplicate average) was normalized to the detection level of the internal control β-actin house-keeping gene (Pre-developed assays, PDAR, Applied Biosystems, Rotkreuz, Switzerland). Expression data were calculated, as referred to β-actin gene expression in each sample, by using the $2^{-\Delta {\text{C}}_{\text{t}}}$ method [22]. For all genes analysed, dynamic linear range of expression-detection was consistent at least up to Ct value of 35 which was therefore considered as the cut-off of significant values. A threshold of 2-fold increase in specific gene expression over control values was considered as cut-off for the definition of positive responses.

### Statistical analysis

All statistical analyses were performed by using SPSS 15.0 software for Windows (SPSS Inc. Chicago, IL, USA). Correlations between the expression of different cytokine genes and MHC-multimer staining or antibody titres were evaluated by the Kendall's tau correlation coefficient (r) and data were considered statistically significant in the presence of p < 0.05. The significance of differential gene expression in paired samples at different days after influenza vaccination was analyzed by the non parametric Wilcoxon signed rank test.

## Results

### Detection of antigen specific responses from limiting numbers of T cells in whole blood by qRT-PCR

In initial studies we addressed the possibility of using qRT-PCR technology coupled with RNA extraction from WB samples to magnify antigen specific immune responses from low numbers of T cells. To provide reliable quantitative assessments, we spiked cells from a HLA-A0201 restricted CD8+ CTL clone recognizing gp100_280–288 _melanoma associated epitope in allogenic WB from a HLA-A0201+ healthy donor and we incubated the mixture for 5 hours in the presence of a 10 μg/ml final concentration of specific or control (Melan-A/MART-1_27–35_) peptide. Total cellular RNA was then extracted, reverse transcribed and amplified in the presence of primers and probes specific for β-actin house keeping gene and genes encoding different cytokines.

**Figure 1:**
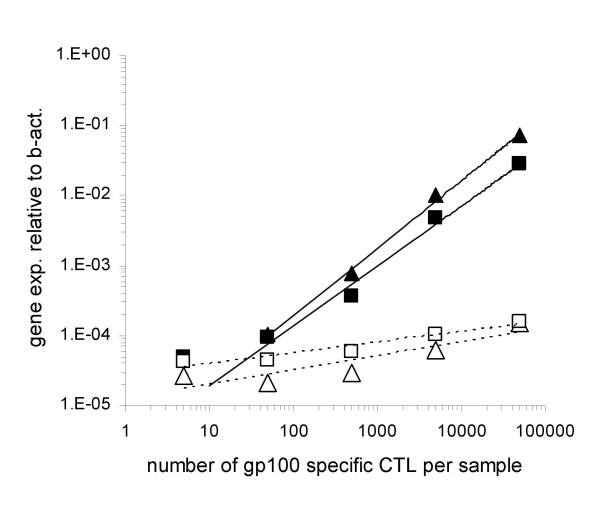
**Monitoring of CTL spiking by WB technology**. CD8+ T cells from an HLA-A0201 restricted gp100_280–288 _specific CTL clone were added to 300 μl WB from an unrelated donor in the presence of the specific or a control (Melan-A/MART-1_27–35_) peptide at a 10 μg/ml concentration. Following 5 hour incubation at 37°C, RNAlater was added to the samples and total cellular RNA was extracted, reverse transcribed and amplified in the presence of primers and probes specific for IL-2, IFN-γ. The expression of the indicated genes from triplicate samples was analyzed by using, as reference, the expression of β-actin house keeping gene (y axes). Standard deviations, never exceeding 5% of the reported values were omitted. A threshold of 2-fold increase in specific gene expression over control values was considered as cut-off for the definition of positive responses. Numbers of CTL spiked into WB were reported on x axes. (triangles = IFN-γ gene; squares = IL-2 gene; filled symbols = specific peptide stimulation; empty symbols = control peptide stimulation).

Expression of IFN-γ and IL-2 genes was significantly (p < 0.05) increased in cultures performed in the presence of specific, as compared to control peptides (figure 1, panel A), thus ruling out the possibility of a prevailing allospecific responsiveness from host WB T cells. In line with these data, the extent of the increased expression of these genes was strictly dependent on the number of spiked gp100_280–288 _specific CTL. Most importantly, these results indicate that specific antigen stimulation provides an activation signal detectable 4.8-fold and 2-fold above background for IFN-γ and IL-2, respectively, in WB down to a minimum concentration ≤50 CTL in a 300 μl sample, thus suggesting that qRT-PCR monitoring of antigen specific immune responses in WB is feasible with a sensitivity comparable to that of qRT-PCR monitoring in ficoll isolated PBMC [8].

### Stability of WB RNA preparations

RNA stability might of decisive relevance in the performance of qRT-PCR and critically affect immune monitoring methods based on the analysis of cytokine gene expression, particularly in the context of field studies. Thus, we stored antigen stimulated WB samples supplemented with "RNAlater" at room temperature, at 4°C or at -20°C for one week prior to gene expression analysis. We observed that no significant variations of specific signal were detectable in whole blood samples stored in the different conditions under investigation (data not shown).

### Responsiveness to virus derived HLA-class I restricted epitopes in whole blood

Based on these studies we attempted the detection of cellular immune responses directed against HLA-class I restricted epitopes derived from viral antigens. WB from two different HLA-A0201+ seropositive donors was incubated in the presence of HCMV pp65_495–503_, or EBV LMP-2_426–434 _and EBV MLF-1_259–267 _virus derived epitopes at a 10 μg/ml final concentration. A well characterized HLA-A0201 restricted influenza matrix (IM) _58–66 _peptide was also used at the same concentration. Moreover, in order to further support the specificity of the WB assays, we comparatively evaluated in the same amounts of WB multimer staining and cytokine gene expression upon peptide stimulation.

**Figure 2:**
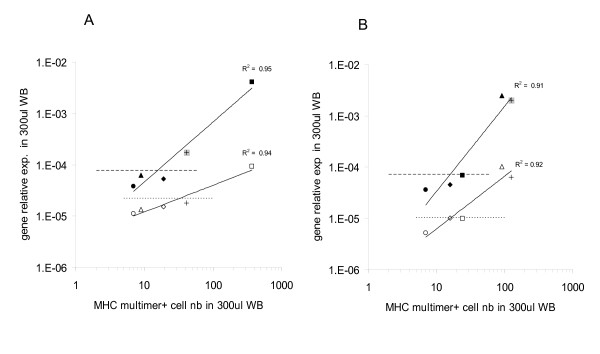
**Cytokine gene expression induced by HCMV, EBV and influenza virus derived HLA class I restricted antigenic peptides in WB of healthy donors**. WB from two HLA-A0201+ healthy donors (panels A and B), seropositive for HCMV and EBV (300 μl) was incubated for 5 hours in the presence of HCMV pp65_495–503 _(triangles), EBV LMP-2_426–434 _(diamonds), BMLF-1_259–267 _(squares) and IM_58–66 _virus (crosses) derived peptides at 10 μg/ml final concentration. Melanocyte derived GP100_280–288 _peptide (circles) was used as negative control. RNAlater was then added and total cellular RNA was purified, reverse transcribed and amplified in the presence of primers and probes specific for IFN-γ (full symbols) or IL-2 (empty symbols). Specific gene expression was analyzed by using, as reference, the expression of β-actin house keeping gene (y axes). A threshold of 2-fold increase in specific gene expression over control values was used as cut-off (dashed lines for IFN-γ and dotted lines for IL-2 gene expression, respectively). WB specimens of the same size (300 μl) from the same donors were simultaneously stained with the corresponding multimers and the number of antigen specific T cells (x axes) was evaluated and correlated with antigen driven gene expression data.

Data from the two donors are reported in figure 2, panels A and B. In both cases a highly significant correlation was observed between the level of IL-2 and IFN-γ gene expression (r = 0.854 p = 0.001 and r = 0.629, p = 0.012 respectively) induced by HCMV pp65_495–503_, EBV BMLF-1_259–267_, EBV LMP-2_426–434 _and IM_58–66 _HLA-A0201 restricted peptides and the numbers of CD8+ T cells stained by specific multimers in the same amount of WB (300 μl). Notably, a 4.5-fold increase in IFN-γ gene expression in IM_58–66 _stimulated, as compared to control WB from the donor depicted in figure 2 panel A, was observed in the presence of only 41 CD8+ cells staining positive for the specific multimer, thus confirming spiking data (see above).

### WB monitoring of HBsAg specific cytokine gene expression in healthy donors vaccinated against HBV

**Figure 3:**
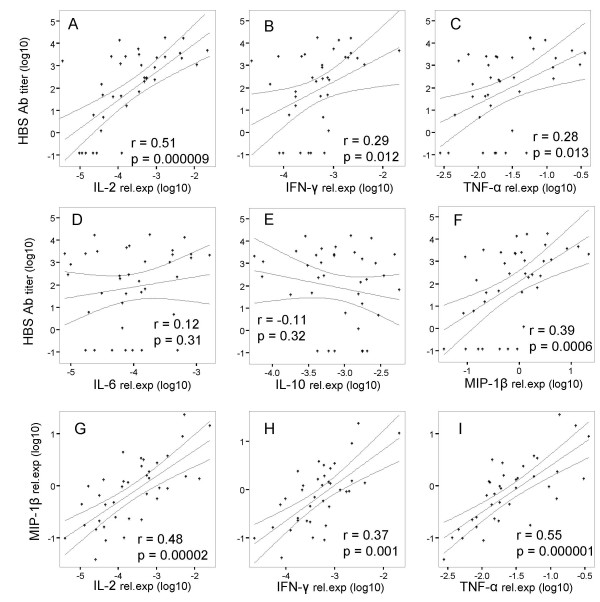
**Correlation between expression of genes encoding cytokines and chemokines and anti HBsAg serum titers in vaccinated healthy donors**. WB from donors naïve or vaccinated with HBsAg was incubated o/n in the presence of a 2 μg/ml concentration of HBsAg. Following addition of RNAlater, total cellular RNA was extracted, reverse transcribed and amplified by qRT-PCR in the presence of primers and probes specific for the indicated genes and β-actin house keeping gene (panels A-F). Cytokine and chemokine gene expression was evaluated by using, as reference, the expression of β-actin gene, as detailed in "materials and methods". Titers of anti HBsAg antibodies were measured by ELISA. Data regarding correlations between expression of MIP-1β (CCL4) and IFN-γ, IL-2 and TNF-α genes are shown in panels G-I. Linear regressions and 95% mean prediction intervals are reported in each panel.

Data regarding cytokine gene expression in WB from spiking experiments or upon stimulation with peptides derived from viral antigens suggested the feasibility of a sensitive WB monitoring of cellular immune responses. Validation of this technology, however, requires comparison with known clinical end points or accepted surrogate markers. Thus, we comparatively analyzed cytokine gene expression induced in WB by hepatitis B virus surface antigen (HBsAg) and specific antibody titers in healthy donors (n = 29 for a total of n = 39 samples) vaccinated against Hepatitis B virus. Samples from naïve, seronegative donors were also studied (n = 9). WB specimens were cultured o/n in the presence of a commercial vaccine preparation (see "materials and methods") diluted to a final HBsAg concentration of 2 μg/ml. We found a highly significant correlation between antigen stimulated expression of IL-2 gene as detectable by the WB assay and specific antibody titers (r = 0.50, p = 0.000009) (figure 3, panel A) in donors vaccinated between six months and twenty years before the tests. Expression of IFN-γ and TNF-α genes was also significantly, albeit not as strikingly, correlated with specific antibody titers (r = 0.29, p = 0.012 and r = 0.28 p = 0.013, respectively) (figure 3, panels B and C). HBsAg induced IL-2 gene expression was also highly significantly correlated with IFN-γ and TNF-α gene expression (r = 0.50, p = 0.0000085 and r = 0.44 p = 0.0001, respectively). Confirmative tests performed on purified T cells showed that the expression of these cytokine genes was mainly due to CD4+ T cell activation (data not shown).

In contrast, expression of IL-6 or IL-10 genes upon HBsAg stimulation was modest and neither correlated with specific antibody titers nor with each other, nor with IL-2, IFN-γ and TNF-α gene expression (figure 3, panels D and E).

Comparative assays were performed with samples from two seropositive and one seronegative donor. HBsAg was able to induce IL-2 and IFN-γ gene expression in cells from seropositive donors only. However, no antigen specific lymphoproliferation or cytokine production, as detectable by ELISPOT could be observed in any of the donors under investigation, suggesting that WB qRT-PCR monitoring may be endowed with a higher sensitivity, as compared to these techniques.

In an effort to identify additional markers of cellular immune response in WB correlating with HBsAg specific antibody titers, expression of chemokine genes was also evaluated. We observed that HBsAg stimulated expression of MIP-1β (CCL4) gene was highly significantly correlated with specific antibody titers (r = 0.39, p = 0.0006) (figure 3, panel F). Notably, the extents of IL-2 and MIP-1β gene expression induced by HBsAg were also highly significantly correlated with each other (r = 0.48, p = 0.00002). Furthermore, MIP-1β gene expression was highly significantly correlated with IFN-γ and TNF-α gene expression as well (r = 0.37, p = 0.001 and r = 0.48, p = 0.00001, respectively, figure 3, panels G-I). Thus, WB monitoring technique helped defining a novel gene expression profile significantly correlated with protection against HB infection.

### WB monitoring of cellular immune response to influenza vaccine: a longitudinal study

**Figure 4:**
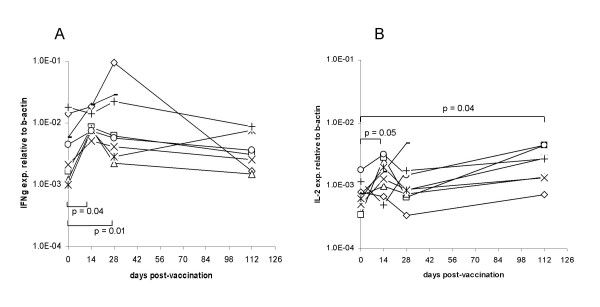
**WB monitoring of cellular immune response to vaccination against influenza virus**. Eight healthy donors were vaccinated against influenza virus. WB specimens were obtained before vaccination (day 0) and 14–112 days afterwards. WB samples (300 μl) were incubated o/n in the presence of a 0.6 μg/ml concentration of influenza hemagglutinin. Values related to the expression of IFN-γ (panel A) or IL-2 genes (panel B) were calculated by using, as reference, the expression of β-actin house keeping gene (y axes).

These results stemmed from experiments performed at single time points. In order to further validate the WB protocol proposed here, a prospective longitudinal study aimed at the monitoring of cellular immune response to influenza virus specific vaccination (winter 2007) was then performed. WB from healthy donors (n = 8) obtained prior to influenza vaccination and at different time points, 2–16 weeks after it, was cultured o/n, as detailed above, in the presence or absence of a commercial vaccine preparation (see "materials and methods") diluted to a final concentration of influenza hemagglutinin of 0.6 μg/ml. Total cellular RNA was then extracted and reverse transcribed and cytokine gene transcripts were amplified in the presence of specific primers and probes. Interestingly, significant increases in antigen specific IFN-γ gene expression as compared to pre-immunization values were detectable at two and four weeks after vaccination (p = 0.04 and p = 0.01, respectively), declining thereafter. At four months after vaccination, however, levels of antigen stimulated IFN-γ gene expression were back to pre-vaccination values. Notably, antigen specific IL-2 gene expression displayed a trend (p = 0.05) towards increased values in the weeks following vaccination and still showed a significant (p = 0.04) responsiveness at four months after administration of the vaccine (figure 4, panels A-B).

## Discussion

Monitoring of cellular immune responses still represents a challenge. Usually, relatively advanced cell culture skills and sophisticated equipment are required. Furthermore, current techniques are difficult to standardize [5,23], also due to the use of biologicals of different origin, e.g. human sera or FCS or to the differential sensitivity of detection equipment. Importantly, vaccination campaigns necessitating accurate monitoring of cellular immune response in cohorts of individuals are sometimes conducted in regions where laboratory facilities are inadequate, if at all available.

In this work our aim was to design and test a RT-PCR based technique easily amenable to standardization and automation for the monitoring of cellular immune responses in WB, simple enough to be performed, at least in its initial steps, by personnel with basic laboratory training, utilizing widely available equipment.

Indeed, similar techniques have already been used to monitor responsiveness to bacterial products, and, in particular, to LPS [14,24]. Furthermore, allospecific immune responses have also been assessed by qRT-PCR in whole blood [15,16] and responsiveness to allergen stimulation has been explored by testing IL-4 gene expression in whole blood [13], predominantly attributed to circulating basophils.

However, the correlation of specific profiles of cytokine gene expression with markers of protection against infection has not been attempted so far, thus preventing a reliable assessment of the potential clinical relevance of this technology in a clinical setting.

Here we describe a technique capable of detecting antigen specific cellular immune responses with a sensitivity and specificity matching that of current technologies requiring PBMC isolation. Its application allows the detection of functional activities in limited numbers of cells. Notably, both CD4 and CD8 specific responses can be reliably evaluated.

The examples provided by our study suggest that this method might qualify as technology of choice for a number of different applications. On one hand, it might prove particularly important in "field" immunization studies with difficult access to laboratory facilities. Indeed, although the subsequent analysis of cytokine gene expression requires adequate infrastructure, initial antigen stimulation and safe storage and transportation of test probes can be performed with minimal equipment and time requirements. Furthermore, automation of this method could be advantageously utilized for fast and accurate quantitative monitoring of natural or vaccination induced cellular immune responses in large groups of vaccinated individuals.

On the other hand, the power of this method as discovery tool should also be underlined. Our data document for the first time that in vaccinated individuals the capability to express MIP-1β (CCL4) gene in response to HBsAg is highly significantly correlated with specific antibody titers. Indeed, this chemokine has been suggested to play an important role in antiviral defense, either by direct mechanisms or following the activation of cells presenting viral antigens to T cells [25-28].

Taken together our results indicate that WB antigen specific stimulation of cytokine gene expression could emerge as an important tool for the screening of cellular immune response to large panels of antigens or peptides and the rapid identification of novel antigenic epitopes. Classical methods allowing the physical identification and the sorting of cells endowed with peculiar functional profiles could then be used to address the precise characterization of antigen specific T lymphocytes in selected subpopulations of donors.

## Competing interests

The authors declare that they have no competing interests.

## Authors' contributions

ES-T and NR performed qRT-PCR assays in WB, DMF designed the study and participated in the performance of qRT-PCR assays and in writing the paper. DM helped collecting samples from HBsAG vaccinated donors and performed serological studies. CF-M helped designing the qRT-PCR strategy. GCS provided funding and helped designing the study and writing the paper. PZ designed the qRT-PCR strategy, evaluated the gene expression data and wrote the paper.
